# Parasite Survival and Disease Persistence in Cystic Fibrosis, Schistosomiasis and Pathogenic Bacterial Diseases: A Role for Universal Stress Proteins?

**DOI:** 10.3390/ijms221910878

**Published:** 2021-10-08

**Authors:** Priscilla Masamba, Abidemi Paul Kappo

**Affiliations:** Molecular Biophysics and Structural Biology (MBSB) Group, Department of Biochemistry, Kingsway Campus, University of Johannesburg, Auckland Park 2006, South Africa; akappo@uj.ac.za

**Keywords:** cystic fibrosis, *Escherichia coli*, schistosomiasis, tuberculosis, universal stress protein

## Abstract

Universal stress proteins (USPs) were originally discovered in *Escherichia coli* over two decades ago and since then their presence has been detected in various organisms that include plants, archaea, metazoans, and bacteria. As their name suggests, they function in a series of various cellular responses in both abiotic and biotic stressful conditions such as oxidative stress, exposure to DNA damaging agents, nutrient starvation, high temperature and acidic stress, among others. Although a highly conserved group of proteins, the molecular and biochemical aspects of their functions are largely evasive. This is concerning, as it was observed that USPs act as essential contributors to the survival/persistence of various infectious pathogens. Their ubiquitous nature in various organisms, as well as their augmentation during conditions of stress, is a clear indication of their direct or indirect importance in providing resilience against such conditions. This paper seeks to clarify what has already been reported in the literature on the proposed mechanism of action of USPs in pathogenic organisms.

## 1. Introduction

Over countless centuries, several organisms have mastered the art of surviving a wide array of adverse conditions (also known as stressors) such as temperature, pH, radiation, salinity, toxic chemicals, nutrients, and low oxygen levels, which are able to modify the homeostasis and status of cells [1]. The adaptation and survival of various organisms under stressful conditions owe part of their success either to the augmentation of regulatory and protective stress resistance mechanisms or to the down-regulation of nonstress genes. Among the former exists an enigmatic family of proteins, known as universal stress proteins (USPs). They are recognized as a conserved family known as the universal stress protein A (Pfam classification PF00582) orthologous superfamily (COG0589) [2]. Their name describes the nature of their function; these proteins are involved in cellular responses to abiotic and biotic stresses alike that include, but are not limited to, heat shock, acid and/or high salinity environments, the presence of oxidants, DNA damaging agents, macromolecular damage, nutrient starvation, uncouplers, and pH and antibiotic stress, while they physiologically function in cell growth and development regulation, ion scavenging, cellular mobility and hypoxia responses [2,3,4,5,6]. The universal stress protein, first known as C13.5, was discovered more than 25 years ago in *E. coli* as a characteristic 13.5 kDa cytoplasmic protein that appeared as a unique spot on a two-dimensional isoelectric focusing PAGE-gel system by Nystrom and Neidhardt in response to some of the above-mentioned stresses, apart from cold shock and the tetracycline antibiotic, which failed to elicit the response [7,8]. At the time, several global regulators were under study and it was known that most proteins encoded by stress or starvation stimulons were generally induced by a specific stimulus although stimulons could share member proteins. UspA was unique because it was a universal non-specific responder to various stimuli. Subsequent studies suggested that UspA plays a role in both protection and regulation as it pertains to growth arrest due to UspA-deficient *E. coli* strains that struggled to survive extended periods of growth inhibition triggered by several stressors [9]. Other studies suggested that the production of UspA was more growth phase related than growth-rate dependent because growth-arrested cells focus more on maintaining their current state, using the resources that remain after depletion rather than on proliferation and growth [10,11]. The importance of this protein for cell survival was demonstrated in several ways: (1) production of mutant *E. coli* cells lacking UspA show increased susceptibility to death or impairment when exposed to complete and prolonged growth inhibition stresses such as extended carbon starvation, H_2_O_2_, carbonyl cyanide chlorophenylhydrazone, cadmium chloride and dinitrophenol exposure, as well as osmotic shock; (2) the over-expression of UspA results in the blockage of growth-arrested cells; and (3) mutations in the *uspA* gene exert an effect on gene expression timing, which in turn accelerates gene expression pattern changes and protein synthesis [9,12,13]. Transcription of the *uspA* gene is a result of a σ70-dependent promoter, which is turned off rapidly once a nutritional up-shift is observed in growth-arrested bacteria [13,14]. The fact that the gene is incorporated in the FadR regulon suggests its potential role in fatty acid or membrane lipid metabolism, since FadR represses and activates genes involved in fatty acid degradation and biosynthesis respectively [7,10]. Positive regulation of the *uspA* gene occurs via alarmone guanosine tetraphosphate or ppGpp, a key nucleotide that regulates numerous stationary phase-induced genes, which it does through the β-subunit of RNA polymerase [7,11]. The UspA protein is a serine and threonine autophosphorylating protein whose phosphorylation is highly dependent on the TypA tyrosine phosphoprotein, which is activated in response to stasis [10,15]. A possible explanation for the importance of this feature is the ability of UspA to regulate the detrimental effects of other proteins in growth-arrested cells. It was suggested that UspA in coercion with the o591 tyrosine phoshoprotein degrades such proteins by phosphorylation tagging [10]. Some studies also suggested that the increases in UspA phosphorylation may be the factor responsible for the acidification of a minimum of six proteins during the early stages of growth arrest in cells [10,16]. Other studies suggested that the absence of this protein increases sensitivity when cells are exposed to mitomycin C and UV irradiation, since it forms part of the RecA-dependent DNA protein and repair system [17].

**Figure 1:**
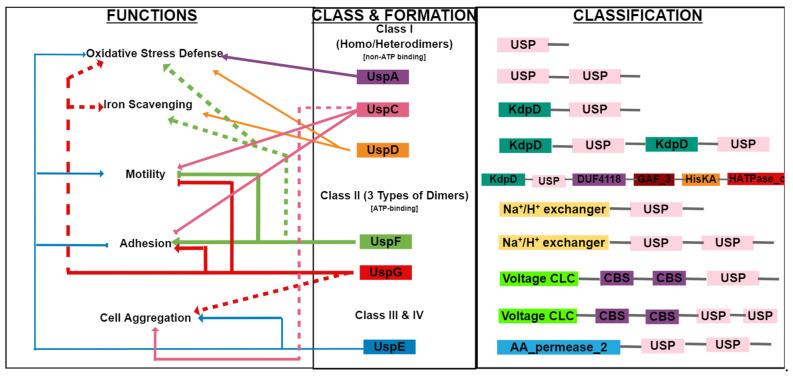
Classification, domain composition and functions of known USPs. The figure depicts the multifaceted but overlapping functions of USPs and the form in which they exist in nature.

Subsequent analysis of the *E. coli* genome led to the discovery of five more usp family genes in bacteria, *uspC*, *uspD*, *uspE*, *uspF,* and *uspG*, which are all associated with adhesion, motility, and resistance to oxidative stress [15,18]. Together with *uspA*, *uspD* not only defends against agents responsible for superoxide generation but is also a regulator of intracellular iron. *uspE* and *uspD* on the other hand, are essential for cellular motility, which takes place at the expense of cell adhesion through *uspE* and *uspD* genes, whose link to cell adhesion has been demonstrated via electron microscopy. However, *uspF* and *uspD* exhibit the exact opposite of *uspE* and *uspD* effects [15,19,20]. All this proves the evolvement of USPs to exhibit various physiological roles within the cell for its survival, defense and escape from cellular stress (Figure 1) [15].

Since then, through the sequencing of genomes, the presence of universal stress proteins have not only been located in bacteria, but were observed to be widely distributed in archaea, plants, metazoans, as well as in yeast, fungi, invertebrate, and protist genomes, forming a significant part of their defense systems against various forms of stress [17,18,21]. The presence of USPs has also been observed in various diseases, mostly in the pathogens responsible for them. For the most part, a significant component of pathogen virulence to the host is dependent on stress resistance [17,21]. This is because colonization and pathogenicity in the host largely depends on overcoming various host responses, such as depletion of nutrients and the presence of reactive oxygen species. The existence of USPs in certain microorganisms has shown that they are involved in contributing to pathogen colonization as well as the establishment of persistent infections in humans. Examples of these include USPs in *Acinetobacter baumannii*, *Salmonella typhimurium*, as well as Usp isoforms in *Mycobaterium tuberculosis* and in various strains that cause cystic fibrosis (CF) and schistosomiasis [3,22]. Although the importance of USPs has been highlighted in the literature and a considerable amount of work has been done in studying the regulation and physiology of these proteins, the molecular mechanisms by which they provide protection and stress resistance remain elusive [3,22,23]. This paper attempts to draw more attention to this ubiquitous and widely distributed group of proteins by elucidating proposed molecular mechanisms in context of their contribution to pathogen survival and infection persistence.

## 2. Structure of the USP Family

Most of the information on the biochemical and physiological attributes of Ups stem from being studied in *E. coli* [24]. The USP domain is a highly conserved 140–160 amino acid-containing domain that may exist in one of three forms: as a single 14–15 kDa domain that makes up the whole protein, as a tandem domain of approximately 30 kDa within larger USP proteins on a single polypeptide chain, or as two Usp domains fused together with catalytic domains that include Cl^−^ voltage channels, Na^+^/H^+^ antiporter domains, protein kinase domains or amino acid permeases (Figure 1) [15,25,26]. A large contributing factor to the ability of USPs to resist multiple stresses is the structural diversity associated with the functional motifs attached to the USP domain caused by the process of evolution, which may have resulted in the attachment of these motifs that hold various biochemical and molecular consequences [3]. *E. coli* USPs also develop homodimer and heterodimer formations with each other, therefore contributing to the different functional responses they display to a variety of stresses. USPs A, C and D form both homo- and hetero-dimers, while USPs F and G have the ability to form three types of dimers (Figure 1) [21].

USPs are categorized according to their ability to bind ATP; UspA and UspA-like proteins do not bind ATP, while USPs of the F and G-type bind ATP [27,28]. In most cases, USPs that comprise attached domains belong to the FG type. These include USPs F (ynaF) and G (ybdQ) (Class II) as their names suggest, while USPs A, C (yecG) and D (yiiT) (Class I) form part of the UspA subfamily [29]. This subfamily, also known as the USP family (Pfam 00582), falls under the PP-loop clan (CL0039) as categorized by the Pfam database, which consists of 12 members; ATP_bind_3 and ATP_bind_4, Electron transfer flavoprotein, Arginosuccinate synthase, Thiamine biosynthesis protein (ThiI), NAD synthase, Asparagine synthase, ExsB, tRNA methyl transferase, Phosphoadenosine phosphosulfate reductase family, DNA photolyase, and lastly the USPs [14]. The PP-motif exhibits different characteristics among clan members, resulting in a wide range of enzymatic and functional activities. It has been proposed that this domain in turn falls under the α/β domain referred to as the HIGH-signature proteins, Usp-like domains and PP-ATPase (HUP) domains, which is an ancient and diverse group of proteins that contains photolyases, Usps and electron transfer flavoproteins, believed to have evolved from UspA-like proteins by losing their ATPase and nucleotide-binding functions. This has suggested that UspA-like proteins are actually ancestors of UspFG-like ATPase-binding proteins [7,30]. The domains present in this group are mostly small domains assembled as Rossmann fold-like structures consisting of three layers containing five parallel β-sheets with two flanking α-helices on each side [31]. The Structural Classification of Proteins database additionally categorizes USPs under the adenine nucleotide alpha hydrolases-like superfamily, which is in turn described by the superfamily database as possessing general functions, especially in nucleotide transport and metabolism [14]. The class is categorized by having an adenine nucleotide alpha hydrolase-like fold made up of assembled Rossmann fold-like structures consisting of five parallel β-sheets with two flanking α-helices on each side and a three-layer α/β/α core in its interior [14,31]. This fold is in turn placed within the α/β class that is mainly made up of β/α/β units.

Classes III and IV are composed of two UspE (ydaA) units, namely E1 and E2 [6,32,33]. In bacteria, the functions of these classes are multifaceted but may overlap: Class I UspA and D protect against oxidative agents, while UspD is involved in the availability of intracellular iron; Class I UspC and Class II UspF and UspG play their roles in motility and adhesion, while Class III, Class IV and UspE convey overlaying functions with the other classes (Figure 1) [15].

USPs of the FG type contain the ATP binding site that is characterized by the conserved G-2X-G-9X-G-(S/T) motif [6,34]. The binding of ATP serves as a functional regulator of USPs [35]. Leading to this discovery was the study of two crystal structures representative of each sub-family: UspA of *Haemophilus influenzae* and the MJ0577 UspFG from *Methanococcus janaschi*. Similarities have been observed in both their tertiary α/β folds within the asymmetric dimer where adjacent β5 strands result in an anti-parallel interaction forming the interface of both proteins [26,36]. The structural differences notable between the two proteins include their ATP binding activity. A study by Sousa and McKay confirmed that UspA does not bind ATP despite the use of 1 mM MgATP included in crystal growth experiments. Tight ATP binding has however been observed in MJ0557 experiments despite the exclusion of ATP from purification and crystallization experiments. The stimulation of ATP hydrolysis by MJ0577 is suggestive of the protein functioning as a cell factor-dependent ATPase, which sheds more light on the biochemical activities of bacterial USPs. These still require more identification [37,38]. Coordinating the binding of the ATP nucleotide are conserved residues: Gly127 that bind to ribose, Gly130 that binds to β–phosphate and Ser141 that binds to γ–phosphate by hydrogen bonding, respectively, while Gly14 interacts with the α-phosphate and Asp13 and Val14 interacts with the base of ATP [14]. Apart from environmental stressors, the USP up-regulatory response is mediated by the phosphorylation of serine and threonine residues by ATP and guanosine triphosphate, which are both phosphate donors [39]. Instead of glycine amino acids and the ATP binding motif, the UspA of *H. influenza*, on the other hand, contains heavy side-chained residues such as glutamine and methionine. Since it lacks the nucleotide-binding motif, these proteins are likely to form a group of UspA-like ancestors. It is suggested that the two classes evolved so as to perform diverse functions within the cell [14]. Although the exact mechanism of action of USPs is largely evasive, their ubiquitous nature in various organisms, as well as their augmentation during conditions of stress, is a clear indication of their direct or indirect importance in providing resilience against these. Apart from plants, USPs are also present in disease-causing pathogens, therefore studying the structures and functions of these proteins could provide extra opportunities for understanding their involvement in parasite survival and persistent to eventually develop solutions to various diseases. The following sections shine a spotlight on current investigations pertaining to this.

## 3. USPs in Cystic Fibrosis

**Figure 2:**
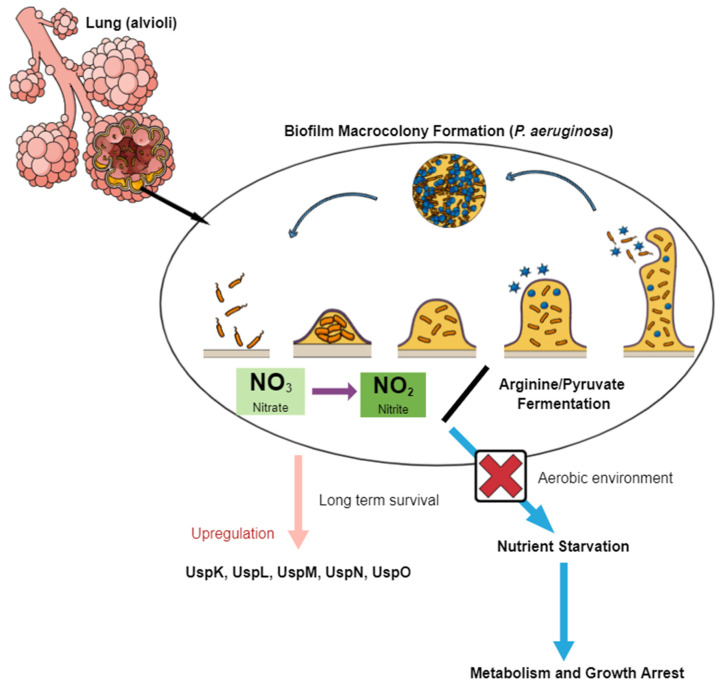
*P. aeruginosa* biofilm formation. Biofilm formation is crucial to the pathogen’s survival, and this can only take place through denitrification, arginine or pyruvate fermentation, otherwise growth and metabolism arrest occurs. Survival is prolonged by the up-regulation of various USPs.

Cystic fibrosis refers to a heterogenous genetic disorder caused by mutations in the CF transmembrane conductance regulatory gene, which encodes the cAMP-regulated chloride channel that eventually develops a lesion in various epithelial cells in the human body [40,41]. This subsequently leads to an array of diseases: pancreatic exocrine insufficiency, biliary cirrhosis, an increase in chloride-concentrated sweat and the most notable, lung disease distinguished by bacterial infection of the sinuses and airways. During infection, dysregulation of various innate immune functions occurs, resulting in the presence of microbial pathogens that cause inflammation and give rise to chronic lung disease accompanied by lung tissue destruction, which are the main reasons for the low life expectancy observed in CF patients [42,43]. Apart from recurrent lung infections, CF is also implicated as a pre-disposer to diabetes, chronic pulmonary infections, gastrointestinal disorders and an array of several other diseases [44]. One of the hallmarks of CF is the formation of a highly anaerobic environment due to limited oxygen levels caused by the presence of colonizing bacteria. Once infection has been established, the respiratory tract is confronted by rises in temperatures of up to 37 °C, pH and nutrient decline, oxidative stress and low oxygen levels that can range from 20.9–0%, due to the presence of anaerobes [45]. *Pseudomonas aeruginosa* is an opportunistic bacterium that has the ability to inhabit various environmental niches and is the main pathogen that contributes to chronic infection in CF patients [46]. The pathogen survives immune attack from the host through the formation of biofilms, leading to high antibiotic resistance and contributing to persistent infection [47]. Data from Worlitzsch et al. showed the pathogen is deeply embedded in stationary mucus, resulting in biofilm-like macrocolony formation, which is typically associated with hypoxia [48]. It grows anaerobically under two main pathways: (i) denitrification via the reduction of nitrate to nitrite, both of which are terminal electron acceptors, and (ii) arginine fermentation (Figure 2) [49,50]. Pyruvate fermentation, on the other hand, allows anaerobic survival but not growth. Therefore, in an aerobic environment without nitrite/nitrate, arginine or pyruvate, the pathogen is exposed to nutrient starvation leading to total arrest of its metabolism and growth [49,51]. Two *P. aeruginosa* genes encoding USP domain proteins have been identified that play a role in supporting long-term survival during pyruvate fermentation and the anaerobic stationary phase known as UspK (PA3309) and UspN (PA4352), respectively [52]. The anaerobic environment is crucial to persistent infection within the CF lung based on the formation of the biofilm-like microcolonies within the lung mucus. Biofilm protects *P. aeruginosa* from host immune responses resulting in increased resistance to antibiotics caused by stationary phase conditions due to the depletion of oxygen. Investigation of the regulation of the two *P. aeruginosa* USPs show that both are significant for successful anaerobic energy stress adaptation and therefore increase survival of the pathogen to specific anaerobic stress conditions. Evidence of this is shown by *uspK* and *uspN* mutants exhibiting premature death, further emphasizing their role in *P. aeroginosa* survival within the anaerobic environment [53].

PA3309 is a Usp-like protein that contains one USP domain and is 37% homologous to *E. coli* UspA. The protein is a 16,196 Da 151 amino acid-containing macromolecule with a 5.31 theoretical pI. PA3309 knockout studies conducted by Schreiber and colleagues showed a 400-fold decrease in anaerobic-long-term survival within 20 days in comparison to the wild type strain and pKS13-complemented strain (contained the PA3309 gene and promoter region) [46]. The study showed that the survival of the pathogen is not only primarily PA3309-dependent during pyruvate fermentation, but it also plays a role in long-term anaerobic energy starvation even in the absence of pyruvate and this was shown in a more profound decrease in the cell numbers of the PA3309 mutant. Regulation of the PA3309 gene is also dependent on an oxygen-sensing regulator known as Anr due to the 4–8 fold increase observed in wild types upon a shift to anaerobic conditions [46]. The Anr regulator is a homolog of the oxygen-sensing Fnr regulator in *E. coli*, which plays a role in mediating adaptation from an aerobic to anaerobic environment [52]. The phenotypes observed during this study showed distinct and non-overlapping functions that are unrelated to universal stress responses [53].

Unlike PA3309, PA4352 is essential during the anaerobic stationary phase and important for anaerobic energy stress survival, therefore playing a crucial role for persistent CF and survival of *P. aeruginosa* within microcolonies in anaerobic mucus plaques [51]. It is also a Usp-like protein that contains two USP domains in tandem. Knockout studies have displayed a decrease in survival during the anaerobic stationary phase due to a lack of nitrate after denitrification and a breakdown in proton motive force [46,53]. Induction of an anaerobic environment restricts production of nitrate and this showed delayed growth and premature death of PA4352 mutants during prolonged anaerobic incubation. A higher decrease in viability in comparison to wild types was observed. The same phenotype was also noticed during a shift from an aerobic stationary phase to an anaerobic environment and this observation was only limited to the absence of the PA4352 gene. A loss in viability more than the wild type was also seen during depolarization of the membrane by the CCCP electron transport chain uncoupler for the induction of energy starvation, demonstrating the protein’s importance in surviving anaerobic energy stress.

Apart from these, three other USPs that are up-regulated during hypoxia via Anr induction have been identified by transcriptome analysis in a microaerobic environment and biofilm-grown cells: UspL (PA1789), UspM (PA4328), and UspO (PA5024) [46,53]. These share similar molecular masses and contain two tandem USP domains respectively. The transcriptional control of these genes is Anr-dependent under anaerobic conditions, like that of UspN and this has been shown from defective mutants although their phenotypic roles have not been identified [52]. In addition, it has been shown that expression of the genes is RelA- and SpotT-dependent, which are two regulators of the stringent response.

*Burkholderia cepacia* complex (Bcc) is an opportunistic pathogen not only present among CF patients but also in immunocompromised individuals, pharmaceutical plants, products and disinfectants, whose eradication becomes difficult to undertake once colonization has been established [54]. *B. cenocepacia*, one of the *B. cepacia* strains, is considered more virulent than *P. aeruginosa* because it is associated with a terminal illness known as “cepacia syndrome” that is typified by high temperatures, septicemia, confluent bronchopneumonia and a general decline in pulmonary function, which leads to patient death within days of infection in comparison to the months after which *P. aeruginosa* patients eventually succumb to it [55,56]. Oxygen concentrations decrease dramatically during CF. The low-oxygen-activated (*lxa*) locus, a novel 50-gene regulon, displays increased transcription under anoxic conditions that is pertinent for *B. cenocepacia* survival and persistence [45]. Deletion of the gene results in the absence of growth in anoxic conditions despite incubation in nutrient-rich media or in minimal media supplemented with glucose as the carbon source. Viability loss is also observed in oxygen-free environments, indicating the importance of the gene in *B. cenocepacia* during oxygen depletion. A study conducted by Cullen and co-workers found that among the 19 proteins that play a potential role in electron transfer, metabolism and regulation encoded by the *lxa* gene elevated during chronic infection, six are USPs and a heat shock protein [57]. All the proteins exhibited an increase (of up to 40-fold) in production during chronic lung infection. Subsequent studies then showed that Usp76 and Usp92 displayed different functions showcasing the functional variety of these proteins [54]. Usp76 was, however, highlighted as the most significant contributor to *B. cenocepacia* survival owing to the changes in phenotype observed in *Δusp76* mutants in comparison to the wild type and its role in protecting against oxidative stress and attachment to host cells in comparison to Usp92. Significant observation of the role of Usp76 in macrophage survival showed its impairment by 80% in the *Δusp76* mutant.

*Staphylococcus aureus* is another major human pathogen that causes various chronic diseases and lung infections, including CF [58]. Successful long-term adaptation strategies comprise the formation of biofilms or small-colony variants, while in the short term the pathogen must adapt to the microenvironment. Using 2D gel electrophoresis to investigate cytoplasmic protein pattern changes, Trefon and colleagues identified the induction and abundance of the putative stress protein (SA1532), also known as UspA, in isolates grown in artificial sputum medium [59]. In accordance with this, other studies also noted up-regulation of the *uspA* gene after six hours of in vivo growth using murine pneumonia models [60]. However, down-regulation of the same gene was observed during initial entry in mouse model airways. The contrast in these observations may result from the emergence of exogenous oxidative stress due to a probable decrease in carbohydrates, which may in turn have triggered increased AA catabolism and an eventual increase in the tricarboxylic acid (TCA) cycle, consequently producing endogenous oxygen radicals. Therefore, without knowing the exact function of USPs, their presence is vital for the long-term persistence of this pathogen.

## 4. USPs in Schistosomiasis

Schistosomiasis is an ancient tropical disease that typically plagues the poorest and most destitute of developing countries, particularly in sub-Saharan Africa, imposing serious economic and social threats on the affected regions [61]. Outcomes of infection include severe cases of morbidity and mortality. In the absence of a vaccine, the discovery of target proteins has been on the agenda for alternative drug discovery to Praziquantel, which is the only chemotherapeutic drug of treatment against the causative agent but is slowly losing credibility because of various side effects and recent evidence of drug resistance. The genome sequence of *S. mansoni*, investigated using whole genome shotgun sequencing, revealed the presence of 11,809 putative genes encoding 13,197 transcripts [62]. This project opened an avenue for the discovery of drug development and vaccine targets, as many of these genes encoded for ligand and voltage-gated ion channels, proteases, lipid metabolism receptors, kinases and neuropeptides. Subsequent researchers realized that eight of these genes, still within the *S. mansoni* strain, are *usp* genes that encode USP domain-containing proteins [8,35]. Six of the genes (Smp_001000, Smp_001010, Smp_031300, Smp_043120, Smp_076400 and Smp_097930) include ATP binding motif residues (thus indicating ATP regulation), a phosphorylation site and ligand-binding residues, while the other two (Smp_136870 and Smp_136890) do not. All the genes are present in at least one of the worm’s life stages, while six of them are more pronounced in the miracidium, which is a crucial stage for transmission of the parasite into the snail intermediate host. It has been proposed that the up-regulation of USPs during the intra-snail stages is triggered by stressors to perform various functions such as protection against oxidative stress induced by hydrogen peroxide within snail hematocytes [35]. Reactive oxygen species are generated by phagocytic cells such as sporocysts and hematocytes in the intermediate host to eliminate pathogens. In the human host, killing of *S. mansoni* schistosomulae coincides with the production of H_2_O_2_ from neutrophils [63]. SAGE profiling showed transcription of Smp_136870 during the miracidium and sporocyst stages, which are both exposed to oxidative stress. This observation suggests that the USP observed may serve as a prime candidate in the defense of these intra-molluscan stages against oxidative stress, which is in turn crucial to the parasite’s survival [35]. Apart from this, Smp_001010 and Smp_043120 displayed the highest transcription in both the intra-molluscan and intra-mammalian stages, highlighting the need for additional studies to be carried out on discovering the specific functions or mechanisms involved in their response to environmental stress.

Mbah and co-workers went a step further and discovered, using bioinformatics-based predictions that there are in fact 13 *Schistosoma* USP sequences, all possessing conserved sites for aspartate, leucine, glycine, histidine and proline at positions 57, 101, 127, 166 and 176 respectively, proposing that these could actually be common regulatory sites for *Schistosoma* USPs [39]. Prioritizing two of the proteins, Q86DW2 from *S. japonicum* and G4LZI3 from *S. mansoni*, three metal ion ligands, namely Ca^2+^, Zn^2+^ and Mg^2+^, were predicted. It was proposed that Mg^2+^ binds to the Mg-ATP binding groove either during phosphorylation or stress responses driven by ATP-dependent mechanisms, thus preventing interaction of the Mg^2+^ ion with ATP at the groove, in turn compromising ATP binding and phosphorylation. In the absence of Mg^2+^, Ca^2+^ simulates ATPase activity, which is crucial, as Ca^2+^ holds important functions in motor and musculature-related activities, egg hatching processes, regulating miracidia to sporocyst transformation and modulating musculature activity, especially during exposure to PZQ. Therefore, it is evident that the expression of USPs in the developmental cycle of schistosomes is imperative for the parasite’s survival and could serve as potential and attractive targets for investigating *Schistosoma* ecology, biology and intervention, especially since USPs are not expressed in humans [8,22].

## 5. USPs of Pathogenic Bacteria

### 5.1. Tuberculosis

Tuberculosis (TB) is an infection caused by a single organism (*Mycobacterium tuberculosis*) and is regarded, alongside HIV/AIDS and malaria, as one of the world’s big three deadliest diseases [64,65]. An estimated 8.9–9.9 million new cases were accounted for in 2008 and this value has not receded, considering the 1.3 million deaths reported worldwide in 2012. It is estimated that at least a third of the world’s population is infected with the causative agent in its latent form. Since 1974, BCG has been the sole vaccine mostly administered to neonates and school children, providing protection of up to 80% in children and 50% in adults [66]. *Mtb* has over 10 USPs that are present during infection and yet their functions remain unidentified [25]. However, researchers have suggested their potential roles in vaccine or drug production, as well as in diagnostics.

**Figure 3:**
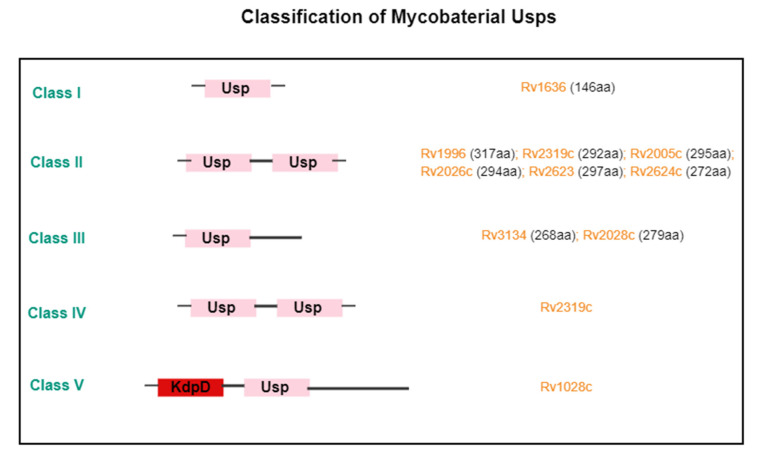
Classification of Mycobacterial USPs. Mycobacterial USPs are grouped into five classes, which altogether contain ten USPs.

The persistence of *Mtb* within the human host in a clinically latent stage for years without causing severe disease, unlike so many other toxic pathogens, is one of the main attributes of this infection’s success [67]. A large contributor to latent TB has been the hypoxic environment. It has been suggested that the limitations associated with the inhibition of *Mtb* growth in vivo is due to reduced levels of oxygen, since TB infection is most often associated with oxygen-enriched sites in the human body. The formation of a granuloma triggered by activated macrophages stunts *Mtb* replication by curbing bacterial access to oxygen and nutrient supply [68]. Ultimately co-infection with other opportunistic diseases, particularly HIV/AIDS, triggers a high chance of developing active disease. There are at least 48 genes under the DosR/DosS/DosT regulatory system that become up-regulated during conditions of nitric oxide stress and hypoxia that play a role in *Mtb* persistence and/or intracellular survival. Of these genes, 44 encode conserved hypothetical proteins and six of these are from the USP family [25]. *Mtb* has 10 USPs altogether, four of which are not regulated by DosR. There used to be eight USPs known to be encoded by *Mtb* H37Rv that were divided into three classes based on domain organization: Rv1636, which is made up of 146 amino acids and contains one USP domain; Rv2028c (279aa), Rv2624c (279aa) and RV3134c (268aa), which all belong to the second class and possess one domain and a second C-terminal domain, and the final class, which consists of two USP domains made up of four USPs: Rv1996 (317aa), Rv2623c (297aa), Rv2005c (295aa) and Rv2026c (294aa) [24]. This has since increased to ten proteins organized into five classes, as shown in (Figure 3) [14]. It has been suggested that *Mtb* USPs are highly related to Actinobacteria [69].

A significant amount of interest is directed at the presence of USPs, the underlying mechanisms involved in their up-regulation and the roles they play in *Mtb* infection, which are very evasive. Many studies have, however, pointed toward their potential use in vaccine design or the development of novel diagnostic tools. One of the most effective methods for preventing TB transmission is early diagnosis. However, available diagnostic procedures display various shortfalls, such as failure to produce detectable sputum and reduced cost efficiency for sputum smear microscopy, as well as their limitations in clinical use due to variable accuracies for serologically based antibody tests [70]. One of the key characteristics of active TB is the activation of the humoral response and the presence of circulating antibodies, which have surprisingly been overlooked as a vaccine target [71,72]. Using proteome microarrays, the combination of Rv2026c and Rv2421c has been identified as a new detectable and reliable biomarker for the diagnosis of active TB [70]. The up-regulation of the *Rv2026c* gene along with three others, *Rv2005c, Rv1996* and *Rv2028c,* has already been shown during *Mtb* hypoxic conditions. Experiments using four *usp* knockout mutants, of which three were DosR-regulated USPs (*Rv1996, Rv2005c* and *Rv2028c*) and the other was the DosR-regulated *Rv2026c USP*, revealed hypoxic survival defects. However, the results seemed to suggest either partial or complete redundancy in USP function in view of the insignificant difference observed in intracellular growth between *usp* mutants and wild types. Explanations for this unanticipated observation span around the fact that the experiments were conducted *in vitro*, suggesting the repetition of this study in models that are more strongly related to in vivo conditions, as well as the biochemical characterization of USPs to explore their actual functions [25]. In addition, the study attributed the results to the differential regulation and overlapping of USP functions, suggesting their necessity once more than one protein is simultaneously knocked out, possibly owing to them serving various functions during stages of infection. The role or significance of Rv2026c and other USPs specifically in *Mtb* infection has been investigated by various authors. Studies highlighting Rv2026c along with Rv2421c (probable nicotinate-nucleotide adenylyltransferase) as two novel biomarkers for TB diagnosis have shown that among other identified antigens, these two exhibited the best diagnostic performance, as they were both recognized by IgG and IgM antibody responses, with Rv2026c exhibiting considerably higher IgG antibody levels in active TB patients in comparison to healthy controls, while Rv2421c yielded higher levels for both antibody levels in active TB patients [70].

**Figure 4:**
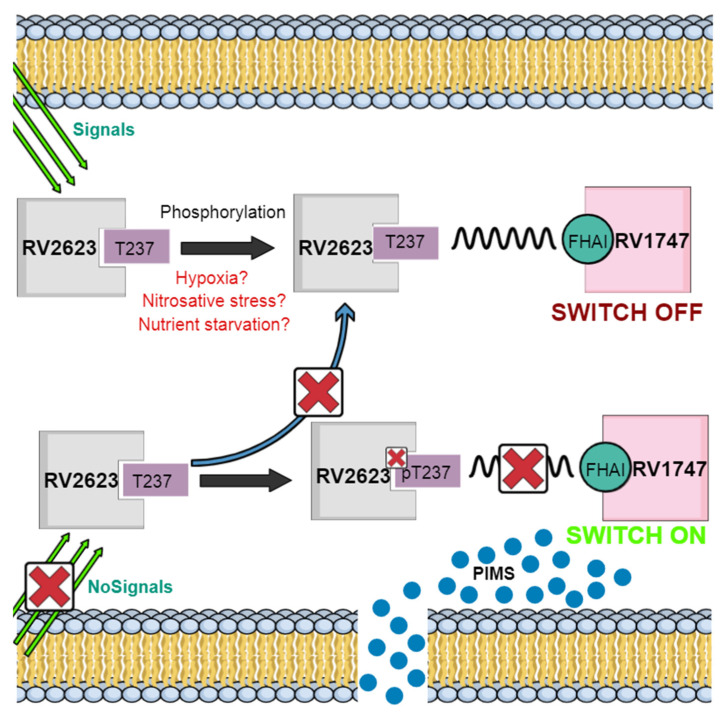
*Mtb* growth regulation by Rv2623. Phosphorylation of Thr237 results in Rv2623 interacting with the FHA I domain of Rv1747 in the presence of certain signals. Without these signals, the interaction between the Rv1747 FHA I domain and Rv2623 is broken due to dephosphorylation, resulting in Rv1747 being “switched off” and thus being able to transport PIMS. Suggestions have pointed toward hypoxia, nitrosative stress and nutrient starvation as possible triggers of Rv2623 phosphorylation, but this remains unconfirmed.

One of the major concerns of TB is the emergence of resistant strains. Studies have shown that the up-regulation of USPs such as Rv1636, Rv2005c and Rv2623 in *Mtb* strains resistant to TB secondary defense aminoglycoside drugs such as Kanamycin (KM) and Amikacin (AM) contribute to *Mtb* resistance. Bioinformatics and proteomic research have shown that Rv2623 over-expresses in AK and KM resistant *Mtb* strains [73]. The Mycobacterium genome codes for both membrane and secretory proteins. Rv2623 is a universal stress protein homolog categorized as the latter that is up-regulated when the pathogen is confronted by inadequate oxygen levels and nitrosative stress, both conditions being initiators of dormancy. Among all the USPs expressed in mycobacteria, Rv2623 elicits most interest because it is one of the most frequently induced stress proteins during hypoxic and nitric oxide (NO) exposure in the dormancy regulon [74]. Mutants deficient in *Rv2623* fail to develop chronic lasting infection in mice, while the overexpression of the protein, on the other hand, augments mycobacterial growth, which indicates the importance of the protein establishing both in vivo and in vitro persistent infection. Mycobacterial growth regulation is ATP-dependent both in vivo and *in vitro*, as the protein contains two USP domains in tandem that are connected by anti-parallel sheets, which altogether possess four ATP-bound nucleotide-binding pockets [74,75]. In conjunction with this finding, an increase in the protein was observed after macrophage phagocytosis as well as in the lungs of infected mice [76,77,78]. More in-depth studies have shown that Rv2623 negatively regulates mycobacterial growth by interacting with Rv1747, a putative ATP binding cassette transporter that exports lipooligosaccharides. Phosphorylation of Thr237 of Rv2623 in the presence of certain signals in the host causes the formation of a phosphothreonine-containing motif, which facilitates interaction between the FHA I domain of Rv1747 and the Rv2623 USP (Figure 4) [79]. This interaction; however, turns Rv1747 off. Without any of the signals from the host or if any other signals arise, Rv2623 becomes dephosphorylated, subsequently leading to its release from Rv1747 FHA I and allowing this protein to transport phosphatidyl-myo-inositol mannosides (PIMs). *Rv2623* mutants have been shown to contain high levels of PIMS in comparison to wild types and *ΔRv2623* is hypervirulent in mice, while the opposite can be observed for *Rv1747* mutants. This suggests the regulation of *Mtb* growth by Rv2623 modulating the export of Rv1747 immunomodulatory PIMS [79].

The up-regulation Rv2005c has been reported in conditions of reactivation and dormancy and further observed in strains that confer KM and AM resistance by somehow deactivating their antibiotic effects, which may eventually lead to extensively drug-resistant tuberculosis [80,81]. Rv2005c possesses two USP domains that fall under the DevR/DosR dormancy regulon, through which it has been suggested to contribute toward resistance through the modification or inhibition of antibiotics. This may also be caused by interactors such as ATPase and glutamine ABC transporter ATP binding proteins that trigger the overproduction of Rv2005c during drug entry via the transporters. In addition, the hypothetical interaction of Rv2005c with other interactors may be involved in respiration, intermediary and lipid metabolism, detoxification as well as virulence. Such investigations uncover new avenues through which this stress protein may be targeted for the development of novel therapeutic drugs to address aminoglycosides resistance.

Mycobacteria possess the remarkable ability to perceive macrophage engulfment, after which the organism is exposed to redox stress that includes superoxide (O_2_^-^), hydrogen peroxide (H_2_O_2_), hydroxyl radicals (OH) and nitric oxide (NO), as well as withstand macrophage attack. Although non-pathogenic, *M. smegmatis* is usually used as a model organism for the commencement of TB research before embarking on studies with *M. tuberculosis*. Exposure of the pathogen to various concentrations of H_2_O_2_ and NO at various time points of infection results in an increase in the expression of DevR/DosR regulon USP proteins such as MSMEG_3940, MSMEG_3945, MSMEG_3950 and MSMEG_5245, which are believed to play a role in restoring redox homeostasis in the cell [82].

### 5.2. Salmonella

It is now a well-known fact that pathogenic bacteria’s survival and colonization are highly dependent on their ability to demonstrate stress resistance and defend themselves against constant environmental insults. Therefore, stress resistance, whether within or outside the host, is imperative for persistence, especially in conditions where nutrient starvation and constant exposure to oxidative radicals are imminent. Studies by Liu and colleagues characterized and illustrated the importance of UspA in *Salmonella enterica* serovar Typhimurium (*S.* *typhimurium*), a major causative agent of diarrheal and acute gastrointestinal disease in patients around the world [83]. About 93.8 million people are affected by human gastroenteritis caused by Salmonella infection, while as many as 150,000 lives are lost as a result of this annually [84]. In vitro experiments suggest that UspA plays a role in the adaptation of this pathogen to metabolic stress and resistance of the organism to oxidative stress [17]. Studies showed an increased expression of the protein in conjunction with UspC during the stationary phase, which is characterized by nutrient deficiency. In addition, UspA protein levels increased during temperature changes. Cells grown in minimal media supplemented with low concentrations of phosphate or glucose had reduced survival rates due to the lack of UspA, while cells exposed to H_2_O_2_ during the exponential and stationary phase exhibited greater survival rates in the latter than in the former, implicating UspA in stress resistance during the exponential phase of growth. In vivo experiments, on the other hand, indicated UspA as an important factor in causing full-blown disease in mice. High inoculum doses exhibited no significant difference between wild types and *uspA* mutants, while low inoculum doses showed decreased virulence in comparison to mutants. It has previously been demonstrated that the absence of UspA results in the premature death of mutants during stasis, while up-regulation induces prolonged growth arrest [9,13,16]. In addition, UspA also demonstrates induction in *E. coli* once exposed to nutrient stress and toxic agents. Further analysis of the *S. typhimurium* genome on stress and virulence genes shows the additional increased expression of UspB, UspC, UspE, UspF and UspG under various pH conditions also during the stationary growth phase and it has been suggested that the presence of these proteins is designed to guard against DNA damaging agents [85]. Although the exact mechanism of action is also not known, the presence of these USPs is imperative to this pathogen’s survival and virulence.

### 5.3. Porphyromonas gingivalis

The oral opportunistic pathogen *Porphyromonas gingivalis* is a Gram-negative anaerobic bacterium of the phylum Bacteroidetes that is predominantly responsible for chronic periodontitis and the expression of various potentially virulent proteases that have the ability to hydrolyze iron-binding proteins, immunoglobins, complement factors and collagen [86]. To survive within the oral cavity and adapt to its variations, which include temperature change, availability of nutrients, the presence of other occupant bacteria or host cells, pH and oxygen tension, *P. gingivalis* requires stress response mechanisms to be able to detect the changes associated with these environmental factors. Studies have shown that apart from heat shock proteins (HSPs), UspA is expressed to assist the pathogen to endure heat stress [87,88]. *P. gingivalis* colonizes different micro-environments and its transition to and from various locations subjects it to environmental stress. A series of stress-related proteins are therefore expressed, most of which are HSPs and Clp proteins. Among these, ClpB and ClpL ATPases act as chaperones whose duty comprise protein remodeling and reactivation. Knockout studies have hypothesized that UspA either compensates for ClpB loss or permits the protein to execute more specific roles in *P. gingivalis* [87]. However, the most notable role of UspA in *P. gingivalis* was demonstrated by Chen colleagues, who revealed the transcriptional and translational up-regulation of UspA during biofilm formation, whose structures are a well-known defense mechanism against environmental stress [89]. Buttressing this was the display of greater resistance by wild type *P. gingivalis* strains to H_2_O_2_ stress and faster growth rates in the presence of unfavorable temperatures and the antibiotic tetracycline in comparison to *uspA*-deficient mutants. The study concluded that the *uspA* gene is an important feature in biofilm development, which plays a role in the resistance of various organisms, including *P. gingivalis*, to environmental stress. Although the mechanism of action for UspA was not defined, it is suggested that the protein performs its role indirectly [90,91]. Concurring with the results of the above study, whole-genome profiling by DNA microarray analysis also noted the conspicuous up-regulation of PG0245, a *uspA* gene induced in response to exposure of *P. gingivalis* to NO stress, suggesting its additional role in NO resistance [90,92]. Consistent with this report was that of Lewis and co-workers, who also detected the elevated expression of the *PG0245* gene in response to a microaerophilic state during the mid-exponential growth phase [93]. Such findings reveal the importance of USPs to the survival of this important pathogen in enduring various microenvironments.

### 5.4. Staphylococcus aureus

Over a third of the world’s population is infected with *Staphylococcus aureus*, a Gram-positive bacterium whose successful infection is a result of its attack on host defense mechanisms [94]. Infection promotes the recruitment of neutrophils by inducing various chemokines and chemokine receptors as well as cytokines, which in turn exhibit antimicrobial defenses such as oxidative killing. However, because of the pathogen’s resistance to antibiotics and its severe virulence, *S. aureus* is responsible for high death rates as well as financial and resource burdens on health care systems [95]. The pathogen is highly adaptable to different environments, colonizing various parts of the human body, most notably causing disorders ranging from mild infection in wounds, deep tissues and the skin, to severe conditions that include septic arthritis, pneumonia, septicemia and endocarditis [96]. The inflammatory response entails the recruitment of phagocytes to the site of infection, resulting in necrosis and fibrin deposits that produce one of the hallmarks of staphylococcal infection, abscesses [97]. Promotion of abscess formation provides the pathogen with a principal defense mechanism and survival tactic by shielding it from the innate immune system in the blood and assuring its persistence in an unfavorable environment. Hence, the up-regulation of endurance proteins produced by the bacteria in the abscess is crucial to ensure *S. aureus* survival. To determine the abundance of such proteins, Attia and colleagues performed proteomic analyses of abscesses from the kidneys of mice infected with *S. aureus*. The results showed the presence of the *NWMN_1600* and *NWMN_1604* genes that encode Usp1 and Usp2 respectively, with Usp2 displaying more expression than the other, which indicates its possible role in host-pathogen interaction. However, comparison between wild type and mutant groups to determine colonization or contribution to pathogenesis displayed no significant difference, suggesting that the abundance of Usp2 within the staphylococcus abscess is not essential for *S. aureus* pathogenesis. The authors suggest that failure to identify the Usp2 role in staphylococcal pathogenesis may either be due to its potential masking by other bacteria in both abscessed and non-abscessed tissues, or the possibility of a more prominent role of the protein in neutropenic mice. Although this may be the case, Usp2 on the other hand, may still play a role in host-pathogen interaction owing to the fibronectin and fibrinogen adhesive fragments the protein possesses [98]. Proteinaceous adhesins, also known as bacterial adhesive proteins, are a large requirement for the development of bacterial infections and USPs are suggested to play a part in this occurrence. *Usp* genes make up part of the 22 genes encoding 24 adhesion-implicated surface proteins, and have been identified, alongside others, to exhibit novel adhesive functions by binding to fibrinogen and plasma fibronectin [99].

Although this is not the first line of treatment, silver has been employed for the treatment of *S. aureus* infection and other pathogenic infections because of its ability to disrupt the bacterial cell wall, resulting in the entry of the Ag(I) ion for the inactivation and disruption of proteins, RNA- and DNA-ases, eventually leading to death. An increase in various stress proteins such as Q2FXL6, a putative USP, was observed in response to stress upon exposure to Ag(I) ions caused by augmented permeability, which may in turn cause oxidative and osmotic stress in the cell [100]. The elevated levels of USPs caused by Ag(I) ion exposure imply its role in assisting the pathogen to mount a defense against stress, which is one of the key features of successful pathogenesis.

### 5.5. Usps of Other Pathogenic Bacteria

Among the organisms threatening the current fight against antibiotic resistance, *Acinobacter baumannii* has emerged as one of the most troublesome for many global health institutions over the last 15 years because of its ability to propel and acquire resistance [101]. In addition, the pathogen is highly susceptible to nosocomial spread owing to its extended persistence in hospital environments. *A. baumannii* usually infects the most critically ill of hospital patients with skin and airway contraventions, leading to detrimental patient outcomes that include pneumonia, bloodstream infections and meningitis [101,102]. UspA, which is highly related to the Usp2 of *A. aureus*, has been identified as an “intriguing therapeutic target” that contributes to *A. baumannii* stress resistance and persistence. During infection, the production of reactive oxygen species such as H_2_O_2_ is inevitable, as this is the host’s first line of defense against invading microbes. Moreover, the ability of the pathogen to persist in unfavorable environments, including those that experience severe pH shifts, requires the pathogen to possess robust survival mechanisms. By investigating the genome of *A. baumannii* for USPs, UspA was identified as the most conserved in all *A. baumannii* strains [2]. Further characterization of UspA observed that expression of the protein in wild types conferred full protection against H_2_O_2_ stress compared to mutants but over-expression of the protein did not confer any additional protection. Other important findings indicated that UspA is essential for *A. baumannii* persistence in low pH conditions, judging from the growth defects observed in mutants. Most notable, however, was the contribution of UspA to virulence and lethality in a murine pneumonia infection model. It was concluded from the results that UspA plays a major role in the pathogenesis of the bacterium and this was particularly interesting, as the Usp2 of *S. aureus* displayed the opposite despite the relatedness of the two proteins [2]. However, more studies are required to determine the exact mechanism at which this occurs.

*Listeria monocytogenes* is a ubiquitous Gram-positive facultative intracellular pathogen largely responsible for foodborne diseases that mostly affect immunocompromised patients who display symptoms of meningitis, neonatal death, abortion and septicemia, as well as pregnant women and their fetuses [103]. Studies have shown that the UspA domain stress proteins convey survival traits to *L. monocytogenes* under its wide variety of stressful conditions that include pH, different temperatures and high osmolarity. Using *usp* deletion mutants and testing *L. monocytogenes* resistance and growth both in vivo and *in vitro,* Gomes and colleagues concluded that the high conservation of USPs within the organism is of importance in the response the pathogen relays to various stress conditions. Mutants showed reduced resistance to low pH and H_2_O_2_ conditions, as well as a decrease in virulence and numbers, while the intracellular transcriptional induction of the *lmo0515*, *lmo1580* and *lmo2673 usp* genes was observed tagging the pathogenic survival of *L. monocytogenes* to the presence of USPs [104]. In vivo studies featuring *G. mellonella* larvae and mice livers and spleens inoculated with *L. monocytogenes* all demonstrated impaired virulence and survival rates of mutants, with the *lmo1580* gene displaying the strongest effects. From this study, the designation of UspL-1 (UspListeria), UspL-2 and UspL-3 has been proposed for the *lmo0515, lmo1580* and *lmo2673 usp* genes respectively within the *L. monocytogenes* species. Other studies using 2D-electrophoresis identified proteins regulated by the alternative sigma factor, σ^B^, and noted an increase in the expression of the Lmo1580 USP, concurring with Gomes and colleagues’ proposition of the importance of the *lmo1580* gene [105]. Induction of the protein was shown to play a role during acid adaptation and stress [105,106].

Antibiotic resistance remains a serious threat to populations around the world owing to bacteria evading the host immune system and its responses, resulting in persistent infections that standard treatment fails to clear up. One of the many ways bacteria escape environmental stress is by entering a non- or slowly replicating physiological state or growth rate [107]. This mechanism is a poorly understood process also known as the non-replicative persistent (NRP) state or dormancy. Examples of NRP models include *M. tuberculosis, M. smegmatis* and *Micrococcus luteus*. Quantitative identification studies within the *M. luteus* strain by Mali and colleagues using liquid chromatography-tandem mass spectrometry identified several proteins related to latency, including UspA. Results demonstrated that only the expression of one of the UspA variants, UspA616 (WP_010079616.1), was up-regulated during the viable but non-culturable state [108]. Alignment of *M. luteus, L. pantarum, M. tuberculosis* and *M. smegmatis* UspA sequences and alignment of the last-named three structures proposed that the *M. luteus* UspA protein consists of two ATP binding Rossman folds that are vital for signaling and nucleotide-binding proteins, thus proposing that the protein carries out its function via signaling and interactions with other stress-related proteins. The relevance of the protein was later confirmed through UspA616 inactivation and competition assays, which revealed clear in vitro phenotypes of drastic viability loss and inability to survive nutrient stress [109]. Recent studies have now shown that in addition, UspA616 plays a critical role in *M. luteus* withstanding both starvation and hypoxia by possibly acting as a molecular switch that causes bacterial survival through the regulation of the TCA cycle and production of glyoxylate shunt proteins such as malate synthase and isocitrate lyase that are up-regulated in the absence of UspA616 [109]. The data in this study therefore sheds light on and provides new perspectives on USPs as molecular switches that regulate the survival of bacteria.

## 6. Conclusions

The constant augmentation of USPs during unfavorable environmental conditions, as well as their restricted presence in eukaryotes, represents a unique opportunity for impeding the presence and survival of organisms that need tolerance to hostile environments. Although their exact function is elusive, it is without doubt USPs play both direct and indirect roles in stress physiology for certain diseases. Therefore, the focus for future investigations should be directed toward determining the physiological and biochemical functions of these proteins in relation to and for advancing knowledge of various disease pathways. Exclusive attention in unraveling functional specificity could also in turn improve insights for the design of new pharmaceuticals.

For a good number of years now, biomedical research has shifted a lot of its attention to discovering and validating novel drug targets by studying proteins and their involvement in disease processes. One of the current major challenges in drug discovery is finding new drugs that cannot only cure diseases, but also improve the quality of life. Most drugs now target proteins and, in some cases, are proteins. Therefore, several studies have been conducted on analyzing proteins to improve insights on the principles of their mechanisms for the design of new pharmaceuticals. The constant augmentation of USPs during unfavorable environmental conditions, as well as their restricted presence in eukaryotes, represents a unique opportunity for impeding the presence and survival of organisms that need tolerance to hostile environments. Thus, the characterization of these proteins warrants exclusive attention to bridge the gap between their various functions and novel drug discovery.

## Data Availability

Not Applicable.
